# A mixed methods study of the postnatal care journey from birth to discharge in a maternity service in New South Wales, Australia

**DOI:** 10.1186/s12913-024-11995-w

**Published:** 2024-12-03

**Authors:** Virginia Schmied, Karen Myors, Elaine Burns, Joanne Curry, Jacqueline Pangas, Hannah G. Dahlen

**Affiliations:** 1 School of Nursing and Midwifery, Western Sydney University, Sydney, Australia; 2 ESSOMENIC PTY LTD https://www.essomenic.net/, Sydney, Australia; 3 School of Public Health and Community Medicine, University of New South Wales, Sydney, Australia

**Keywords:** Postnatal, Postpartum, Patient journey mapping, Midwifery, Length of stay, Community-based care

## Abstract

**Background:**

Service gaps continue in hospital and community-based postnatal care despite a high prevalence of physical and mental health concerns reported by women following birth. The aim of this study was to describe the postnatal journey and the care provided to women and their babies who were at low risk for health complications from birth to discharge from the maternity service.

**Methods:**

A mixed methods design was used to map the postnatal journey, for the woman and baby, from birth to discharge from the maternity service. Data were collected through activity diaries completed by 15 women and telephone interviews with the women two weeks after birth.

**Results:**

The average hospital postnatal stay was 70 h and, in this time, the women received on average, a total of 3 h of direct care from a health professional. That is, 4.3% of the in-hospital postnatal stay was spent interacting with a health professional. Approximately 53 min of care in the postnatal unit was directed at the mother’s health, 50 min on the baby’s health needs, 43 min supporting breastfeeding and 20 min on discharge information. Most reported that hospital based postnatal care was helpful, although they reported that staff on the postnatal unit were rushed and mostly the midwife caring for them was unfamiliar to them. Breastfeeding support in the first 12–24 h was limited, with women wanting more one-on-one access to midwives. Some women received home-based midwifery care, and on average each home visit by a midwife was 29 min. Women who received home-based midwifery care reported that this care was very helpful. Women reported that home-based midwives were more likely to engage women in conversations about their social and emotional needs than hospital-based midwives. All mothers were offered a home visit from a child and family health nurse and most visited a general practitioner in the first week.

**Conclusions:**

Women often experience limited time in direct interaction with midwives in the postnatal unit in hospital. Those who received midwifery care at home were more satisfied with this care, Women are requesting more support from professionals in the early postnatal period.

**Supplementary Information:**

The online version contains supplementary material available at 10.1186/s12913-024-11995-w.

## Introduction

The World Health Organization (WHO) emphasises that the days and weeks following birth – the postnatal period – are a crucial phase in the lives of mothers and babies [1]. Yet globally, postnatal care in hospital is the most neglected aspect of the childbearing continuum [2]. For over a decade, researchers in the United Kingdom (UK), Australia and in other high income countries have reported challenges in providing effective routine postnatal care, both in hospital and in the community [3, 4]. The length of hospital stay following birth has continued to decrease [3, 5, 6]. Some, but not all, women receive home-based midwifery support for one to two weeks after birth, although commonly this only comprises one home visit [5, 7]. Research also demonstrates that in Australia vulnerable mothers, such as young mothers, are less likely to get a home visit [8]. Most women see multiple care providers across the service continuum from antenatal booking to eight weeks after birth and beyond [3, 9]. During the COVID-19 pandemic, many women were discharged from hospital with limited postnatal care, though some positives have been reported such as less visitors and interruption of family bonding time [10, 11].

Furthermore, the transition of care from maternity to community based Child and Family Health Nursing (CFHN) service, health visiting services in the UK and child health nurses in Sweden is characterised by inefficient communication systems, insufficient resources, and limited collaboration between professionals and services [7, 12, 13]. Fragmentation and service gaps continue despite ongoing reports detailing a high prevalence of physical and mental health concerns for women following birth [14–16]. The incidence of severe perineal trauma [17], infection, postpartum haemorrhage [18, 19] and eclampsia [20] have all increased over the past decade. Rising birth intervention also means that increasing numbers of women world-wide are recovering from surgical birth (caesarean sections and instrumental delivery), often requiring additional support and assistance during the perinatal period [21, 22], with increasing reports of birth trauma from interventions [23, 24].

In addition, around 20 percent of women experience anxiety and depressive symptoms both in pregnancy and after birth [25–27] and women who experience five or more physical health symptoms are more likely to experience depression in the postnatal period [28]. Previous research demonstrates that women do not always get the support they need to establish breastfeeding following birth [29] and high numbers of infants receive infant formula prior to hospital discharge [30]. Australian infant feeding surveys demonstrate a dramatic drop in breastfeeding in the first six weeks after birth and only 15% of infants are exclusively breastfed to six months as recommended by the WHO [30].

For over two decades, women have reported a lower level of satisfaction with hospital-based postnatal care [3, 31, 32]. Yet few attempts have been made to understand what women find most beneficial postnatally and the components of care, or the pathways and processes, that currently exist. Moreover, few have aimed to develop and test innovative approaches to improve postnatal care, particularly in hospital. Reports on these models indicate that effective initiatives were those that made changes across the organisation following a continuous quality improvement project [4, 33, 34]. However, generally, evidence-based guidelines for postnatal care have not been implemented in practice, particularly the requirement for individualised care planning.

The aim of this study was to describe the postnatal journey and the care provided to women and their babies who were at low risk for health complications from birth to discharge from the maternity service. In some services in Australia women may receive postnatal care for up to 6 weeks following birth. However, the majority of public maternity services provide midwifery/maternity care for up to 2 weeks postnatal and data indicates that many women are discharged from the maternity service within the first 5 days [5]. In the study site, women were provided with up to two weeks of postnatal care depending on need. The study was conducted just prior to the pandemic in partnership with researchers, policy makers, service managers and clinicians with a view to informing the redesign of postnatal services in one Sydney metropolitan maternity service.

## Methods

This study used a concurrent mixed methods design [35] to map the woman and baby postnatal journey from birth to discharge from the maternity service. Data were collected from 15 women via an activity diary reporting the number and nature of interactions with health professionals in hospital and in the community. The women were also interviewed at two weeks following birth. These data are part of a larger study that also explored the way midwives work in the postnatal unit and the time they spend in interaction with women (unpublished data).

Ethics approval was obtained for all components of the study from the South Western Sydney Local Health District HREC (HREC/14/LPOOL/537).

## Study site

The study was conducted in one Sydney metropolitan maternity unit. The maternity service provides antenatal, birth and postnatal services to over 3,000 mothers and babies annually. In this health district, over 50% of the population were born overseas and 43% speak another language at home. At the time of data collection, the typical LOS in the postnatal unit was between 48 to 72 h. Women who had a vaginal birth stayed in the delivery suite for 2 to 6 h after birth and those who had a caesarean section were transferred to the postnatal unit from recovery. The maternity service also offered up to 2 weeks postnatal care for those that required it and approximately 50% of women received home-based midwifery support. Following discharge from the maternity service, women are referred to the CFHN Service that contacted families with a new baby around 1 to 2 weeks after birth. Families were also asked to take their baby to their general practitioner (GP) at around 7 days post birth for a cardiac check.

### Sample and recruitment

We aimed to recruit 20 women, both primiparous and multiparous, who at 36 weeks gestation, had no current or expected physical or mental health complications. The eligibility criteria also required that participants be able to speak, read and write in English as the diaries were not translated to another language. Posters advertising the study were placed in the antenatal clinic and midwives offered women an information flyer about the study when they attended the midwives’ antenatal clinic for their 36-week visit. In this service, women who have no known physical and or mental health concerns attend the midwife only clinic. Those with physical or mental health concerns are typically seen by a medical professional and or a mental health practitioner as well as a midwife in a specialist clinic. To check eligibility the recruiting research assistant asked women if they or their baby had experienced any health issues in pregnancy that indicated they may require additional postnatal care or services in the community. If a woman indicated they had a physical and or mental health concern, the research assistant explained that they would not be eligible for the study. Women who met the inclusion criteria and who expressed interest were provided with the information and consent form. Once they had signed consent, the research assistant gathered demographic details. A total of 20 women were recruited at 36 weeks gestation.

### Data collection

1. Activity diaries from women
  Women were provided with a paper-based activity diary to record care provided in the postnatal unit and in their home or in the community until they were discharged from the maternity service. Women were guided to start their diaries when they arrived on the postnatal unit. The diaries were organised around hourly time slots and the women were asked to select from a list the activities performed, including who carried out the activity, and for how long (Supplementary file 1). Women were also asked to rate their satisfaction with each interaction and space was provided for open-ended comments. With permission, women were reminded, to complete their diaries, with text messages.
2. Interviews with women
  All women were invited to participate in a telephone interview around 14 days after birth. The interviews were conducted by KM and JP. Women were asked a series of open-ended questions about their experience of hospital and home-based midwifery care or other services, such as what was most helpful, least helpful and how services could be improved (see Table 1). Interviews ranged in length from 15 to 45 min and were audio-recorded transcribed verbatim.

**Table 1:** Questions for interview – 14 days after birth

**Interview (10 to 14 days after birth)**	
1. How are things going for you and your baby since you came home from hospital? 2. Please describe your experience of postnatal care in hospital. (prompts – were the midwives available to you when you needed them; on average how long did midwives tend to spend with you; what day did you go home from hospital – what time of day – how did you find the discharge process? (feeding questions come later) How was the food service in the postnatal ward? Were there times of the day that you could rest? 3. Did you have a midwife visit you at home – please tell me about your experience of midwifery care at home? 4. Have you been contacted by the CFHN service? Will this service be visiting you at home or if they have already visited the mother – What was your experience of the CFHN service? The nurses tend to ask quite a lot of questions – how did you find that? – Did you have all your questions answered? 5. Have you used any other services? Can you tell me about that experience? 6. Can you describe the support you have received in relation to infant feeding in hospital and in the community? Did you attend the breastfeeding education session on the postnatal ward? If so, was that helpful? Some women are seen by the Lactation Consultant in hospital – did you have that service and if so how was it? 7. What was the best thing about the postnatal support you and your baby have received in hospital? 8. What was the best thing about the postnatal support you and your baby have received at home? 9. Are there any aspects of your postnatal care that you were not happy about? Please explain? 10. Can you make any suggestions for improving postnatal care in hospital or at home? 11. Once again thank you very much for using the diary Can you tell me about your experience of using the diary?	

### Data analysis

Data recorded by women in the activity diaries were analysed using descriptive statistics to calculate the length of stay (LOS), the amount of direct care in minutes and hours provided to each mother and baby in hospital and at home; for example, the amount of time provided to women for breastfeeding support. The Essomenic Patient Journey Modelling Tool [36] was used to document / map the data recorded by women in their diaries and this was supplemented by interview data where gaps in service use were noted. Each woman’s journey was mapped using the Essomenic tool and an aggregated map was also produced illustrating the ‘average’ postnatal woman and baby journey (see supplementary files 2 to 6).

Thematic analysis was used to analyse the: qualitative textual data collected through interviews with women and open ended comments in the diaries. Thematic analysis is an iterative process that involves close reading and re reading of data, forming preliminary themes, identifying sub themes and comparing across themes and sub themes to identify relationships [37].

## Results

### Characteristics of women participants

Fifteen of the 20 women recruited completed the diary and made between 10 and 47 entries related to direct interactions with a midwife or other health professional. The remaining 5 women indicated they did not have time to complete the diary on the postnatal unit and withdrew from the study. Fourteen women participated in the telephone interview and one woman indicated she was not available for an interview. Six of the 15 participants were born outside Australia (India (2), Indonesia (1) Macedonia (1), Bosnia (1), Philippines (1)) and all 6 spoke another language other than English. Eight women were primiparous, and all were partnered. Ten had tertiary qualifications, one was a university student, 3 had a trade or certificate qualification. All indicated that they had good family support and that after the birth their partner/husband would help them as well as their mother, mother-in-law or other relatives.

Eleven women had a normal vaginal birth, one had a forceps delivery and three had a caesarean section (two emergency caesarean section and one planned caesarean section). One woman had a preterm birth (35.5 weeks). The majority were well following the birth. Four women had either physical or mental health problems or a difficult social situation, not known at recruitment, that required additional services and support after birth. One baby required readmission to hospital. These differences offered illustrations of alternate postnatal care pathways.

### Postnatal care received

On average women and their babies received 2 h 56 min of direct care from a health professional (e.g. midwife) in the postnatal unit and 6 participants received on average 1 h 40 min of midwifery care at home (see Tables 2 and 3). This time excluded any postnatal care provided in the delivery suite. Women in the study recorded that care during the postnatal unit was provided primarily by midwives. Varying postnatal pathways have been illustrated in the five exemplars of ‘patient journey maps’ provided in supplementary files 2 to 6. Reference is made to these maps in the results below.

**Table 2:** Quantity (min/hr) of In-hospital and home-based postnatal care

	**Mean**	**Range (min/hr)**
**Length of postnatal stay in hospital (15 women)**	70 h	22 – 120 h
**Length of postnatal stay (Vaginal birth (normal & instrumental) 12 women)**	61 h	22 – 120 h
**Total minutes of direct in-hospital midwifery/medical postnatal care**	176 min	80 – 340 min
**Total minutes of direct in-hospital midwifery/medical postnatal care (not including C/S)**	173 min	80 – 340 min
**Minutes of postnatal care in first 12 h in hospital**	25 min (majority monitoring mother and baby on admission)	5 – −65 min
**Minutes of care in 13 to 24 h**	32 min	10 – 90 min
**Minutes of care day 2 including Lactation consultant care**	67 min	15 – 155 min
**Minutes of care day 2 (minus Lactation consultant care)**	56 min	15 – 115 min
**Minutes of care day 3**	53 min	40 – 105 min
**Minutes of care day 4**	38 min	20 – 50 min
**Minutes of care day 5**	37 min	30 – 40 min
**Amount of direct midwifery/medical postnatal care in hospital as a proportion of time spent in hospital after the birth**	4.3%	
**Midwifery care at home**	Mean	Range
**Total number of women receiving midwifery care at home** • **Number receiving 2 home visits** • **Number receiving 4 home visits** • **Number receiving telephone-based care in addition to in person care**	6 women 4 women 2 women 3 women	2 – 4 visits
**Average total minutes of care received in the home (in person and on phone)**	100 min	55 – 140 min
**Average total minutes of care in home in person**	88 min	55 – 140 min
**Average total minutes of telephone-based care to women at home**	24 min	20 – 30 min
**Average length of one home visit**	29 min	20 – 50 min
**Average length of on telephone-based care**	9 min	7.5 – 11 min

### In hospital care

#### How much in-hospital postnatal care did women receive and who provided the care?

The average length of postnatal stay (LOS) across all participants was 70 h. Average LOS reduced to 61 h for women who had a normal vaginal birth (Table 2). The LOS differed based on parity. Primiparous women on average stayed 77.3 h) compared to multiparous women who stayed 51.8 h. The total amount of in-hospital postnatal care provided directly to a woman by a health professional (midwife and medical staff) ranged from 1 h 20 min to 5 h 40 min). Women received approximately 1 h of care each 24 h and this time decreased for those who stayed longer. For example, women still in hospital 4 to 5 days after the birth received between 30 and 40 min of care on those days. Thus, on average, 4.3% of a woman’s time in the postnatal ward was spent in direct interaction with a health professional providing care for her or her baby. This excludes time spent on activities away from, but related to, the woman or baby such as documentation.

Primarily midwives provided in-hospital care and most of the care was provided by a midwife that women considered unfamiliar (highlighted in yellow in Supplementary files 2–6). Women also received care from the obstetric and paediatric junior doctors or registrars primarily to confirm that mother and baby were ready for discharge. Two women received additional consultations from medical staff and one woman had a private obstetrician. Four women (3 primiparous and 1 multiparous) were seen by the Lactation Consultant (LC) to assist with breastfeeding issues. Most women also recorded in their diaries that they used the internet and ‘googled’ a range of websites to seek out information related to mothering and baby care daily while in hospital.

When the midwives leave the room, you often think "Oh, I should have asked that" and then I would probably just jump onto Google and try and find a website that would answer my question, which probably isn't a good thing, but at least you get an idea of what seems to be the norm” (M6, Para 2, interview see Supplementary file 4).

#### What was the nature of the care provided in hospital?

**Fig. 1:**
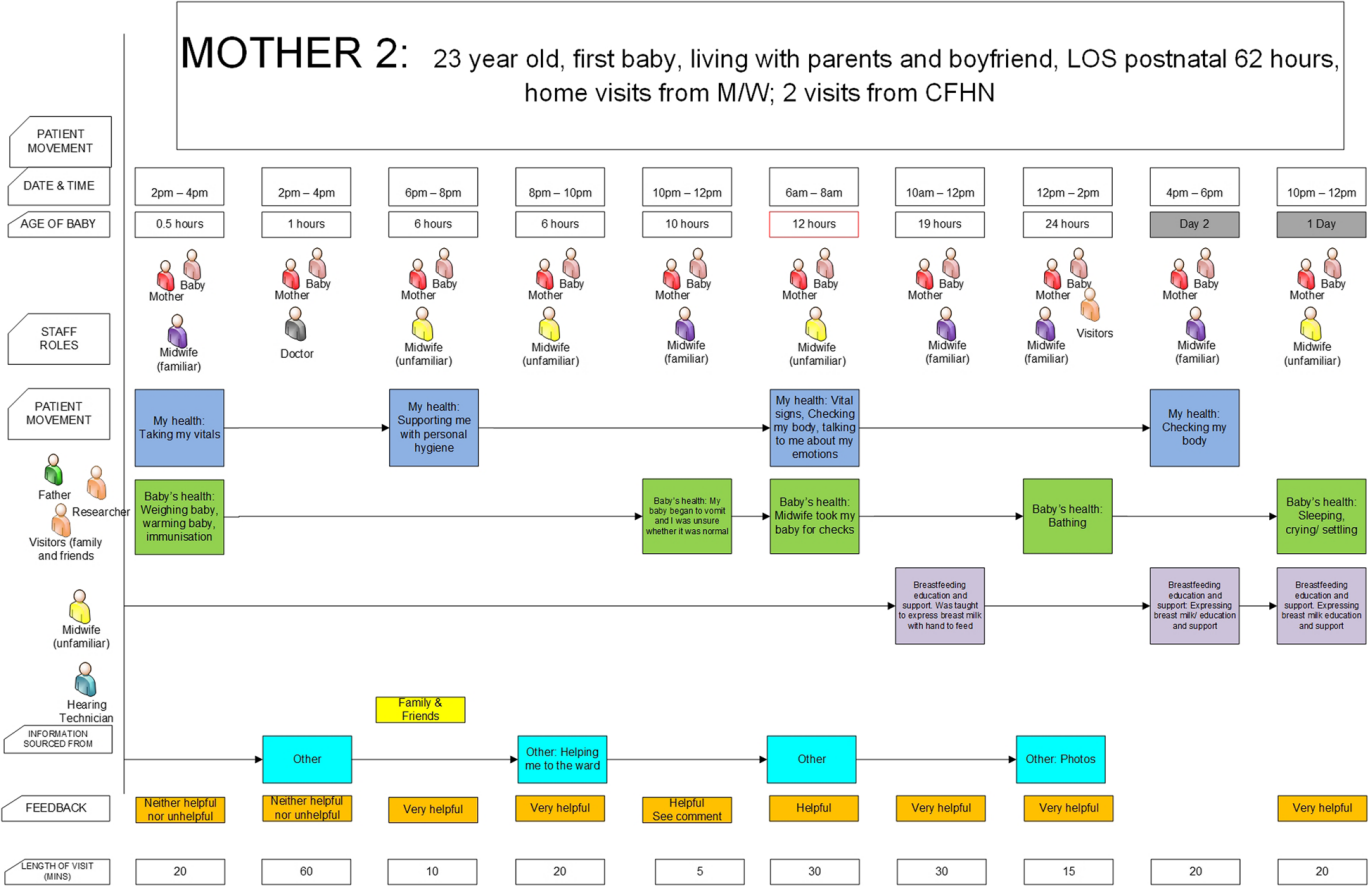
Exemplar: Mother - baby postnatal care journey

Women were asked to identify the care they received from a list of activities (see Supplementary file 1). Figure 1 and Supplementary Files 2 to 6 demonstrate that care activities related to the mother (see the green box in Fig. 1) and baby (see the blue box in Fig. 1) occurred routinely every 12 to 24 h period. Table 3 shows that women received approximately the same amount of care time related to their own health (53 min), and their baby’s health (50 min) across their postnatal stay. Breastfeeding support time (see the grey/pink box in Fig. 1) was 55 min across the full hospital stay which included additional support from an LC received by 4 women. When the LC support was excluded the average amount of in hospital breastfeeding support was 43 min per woman across the full length of stay. There was a difference between Primiparous women who received on average 51 min of breastfeeding support including from and LC and multiparous women who received 29 min on average breastfeeding support. Interactions related to breastfeeding with the LC were longer than those with a midwife (between 20 to 60 min compared with 2 to 10 min). The one woman who was formula feeding did not document any support for infant feeding. Breastfeeding support (see the pink box) occurred least in the first 12 to 24 h and occurred more frequently on days 2 and 3 (see Fig. 1 below).

**Table 3:** Amount of in-hospital care focused on mother’s health; baby’s health; infant feeding and discharge information per women in the study

ID Mother	Parity	Type of birth	Length PN Stay (hr)	Total (min) care*	Mother care (min)	Baby care (min)	Total B/F support (min)	B/F support from LC (min)	B/F support excl. LC (min)	D/C care (min)
M1	P1	Forceps	120	240	70	55	85		85	30
M2	P1	NVD	62	235	52.5	72.5	90		90	20
M3	P1	C/S	105	285	70	60	115		115	40
M4	P1	NVD	64	340	140	70	90	40	50	40
M5	P2	NVD	54	115	34	29	32		32	20
M6	P1	NVD	57	285	62	58	130	80	50	35
M7	P2	C/S	120	200	91	54	45		45	10
M8	P2	NVD 35wk	48	80	45	Nursery	15		15	20
M9	P1	NVD	66	140	38	68	14		14	20
M10	P1	NVD 37wk	96	160	35	35	65	20	45	25
M11	P2	C/S	90	120	40	40	formula		0	40
M12	P2	NVD	29	120	25	25	45	25	20	25
M13	P1	NVD	48	96	30	45	6		6	15
M14	P2	NVD	68	120	30	40	20		20	30
M15	P2	NVD	22	100	25	40	20		20	15
Total			1049 h	2636	787.5	691.5	772	165	607	385
Mean			69.9	176	52.5	49.4	55.1	41.2	43.3	25.7

The apparent gap in breastfeeding support during the first 18 h was noted by several women in their diaries for example:

In the first day, nobody really taught me how to breastfeed. ….I had a midwife come in and say ... "Oh, yeah that seems to be fine," They didn't teach me how, they just saw that the baby was latched on. On day two in the afternoon the lactation consultant came and she told me a different technique (M6, Para 2, diary).

In diaries and interviews, women described the activities undertaken during a postnatal check for example:

I didn't have any postnatal checks during my first 24 hours. The second day, when the nurse [midwife] came she was doing all the checks. She was looking at my stitches because I had some tearing. She was feeling for my uterus, she was asking if I had some pain, she did the basic procedure check and she was asking me how many times I was feeding my baby, how long did I feed, and how often I was feeding. (M11, Para 2, interview)

Women were also asked to record if the midwife asked them about their emotions wellbeing during care interactions. Twelve women recorded that “the midwife asked me about my emotions” but this was the least often recorded postnatal care activity (Supplementary Files 2 to 6). Women reflected on this in their interviews. A couple of women noted they would have liked the midwife to talk more with them about how they were feeling for example:

The midwives were great, they did ask about how I was feeling and asked me about the birth but I would have loved to talk more about how I felt about the caesarean (M5, Para 2, interview).

My baby was in the nursery and no one on the ward ever really asked me how I felt about that. I would liked to have had more time with midwives but I guess they thought the nursery staff talked to me (M7, Para 2, interview).

Observations also indicated that components of the woman’s postnatal check were completed at different times, particularly aspects of the discharge process. Discharge care, which included information on contraception and referral to community services, was typically recorded on the day that the woman was going home from hospital (Supplementary files 2 to 6).

In the diaries, women were asked to rate and comment on the care provided. They generally reported all care activities as helpful. However, women’s text comments were mixed, often indicating they believed the midwives were rushed. Most women described positive experiences indicating that their overall needs were met (see Table 4). Negative aspects of in-hospital postnatal care included having to wait 20 to 30 min for a midwife to answer the buzzer because they seemed to be so busy and that the environment lacked privacy and was too noisy to rest. Several women also gave examples of when they felt neglected by staff.

**Table 4:** Women’s perceptions of in-hospital postnatal care

**Positive Comments**	
They were really helpful. Given how busy they are I was surprised that any question I asked any given time of the day, they were there. (M1, Para 1, diary entry)	
I think probably, being left to our own device to work things out ourselves but still having them close enough to help if we needed it. (M9, Para 1, diary entry)	
There was a lot of support from nurses during night shift. Frequent rounds attended to check baby and mother. Very good interaction. (M7, Para 2, diary entry)	
**Negative comments**	
They were very busy, and you had to wait 20 to 30 min to answer if you called them. (M2, Para 1, diary entry)	
There were no nurses [midwives] who checked patients and baby between 3–9 pm. No education provided. As a mother for 2nd baby there was neglect from nurses [midwives]. (M5, Para 2, diary entry)	
The times I had a midwife in my room were very short and rushed. They would just come in and do what they needed to do but not really ask how I was maybe that was because it was my second baby. (M 11, Para 2 interview).	
The nurse from recovery did not convey messages to midwife or doctors about my stitches from C-section. (M7, Para 2, diary entry)	

These views were reiterated in the interviews and one woman was particularly distressed because she believed the midwives talked down to her:

They treated me like I was stupid. I just tasked a simple question and she rolled her eyes and told me to get on with it (breastfeeding) but I didn’t know what I was doing (M2, Para 1, interview).

### Home-based midwifery care

Table 5 provides a summary of the hours and minutes of midwifery care each woman received at home.

**Table 5:** Quantity of health service provided to or accessed by women in the community

ID	Home-based midwifery	CFHN home visits	Lactation Consultant (LC)
M1		60 m UHHV (day 7)	
M2	60 min over two health visits 22 min over two Phone Calls	90 min UHHV (day 11)	Phone call with ABA (day 10
M3		60 min UHHV & 30 min 2nd home visit (day 9 and 12)	
M4	100 min over four visits 30 min over four P/C’s	75 min UHHV (day 9)	2 hone calls with LC (day 5 and 10)
M5		90 min UHHV (day 8)	
M6		80 min UHHV (day 9) 25 min 2nd home visit (day 12)	
M7		60 min UHHV (day 8)	
M8	90 min over two visits (NICU nurse HV at weeks 2,3)		
M9		60 min UHHV (day 8)	
M10		80 min UHHV (day 8)	
M11	140 min over four visits		
M12	80 min over two visits and 20 min Table 2 wo P/C’s	90 min UHHV (Day 8)	
M13	55 min across 2 home visits	65 min UHHV (day 9)	
M14		60 min sustained nurse home visiting services for young mother)	
M15	90 min over four visits	70 min UHHV (day 9)	

Of the 15 participants, 6 received home-based midwifery support (3 primips and 3 multips). On average, this totalled 100 min of care across 2 to 4 visits in person and in the case of 3 women, an additional 2 to 4 telephone consultations. Mother 8 received 2 home visits from a neonatal nurse when her baby was discharged from the NICU.

It appeared that the offer of community midwifery support was prioritised and matched with need. For example, 2 women having their first baby were both discharged on day 3 (M6 and M9 supplementary files 4 & 5) with no home-based midwifery visits and appeared to be managing well, while M2 and M4 (supplementary file 2 & 3), also first-time mothers were discharged on day 3 and received home-based midwifery care. M2 was 23 years old and was having some difficulties with feeding and M4 had symptoms of anxiety. Common reasons for not receiving home-based midwifery included being outside the catchment area; no positions available on the program; woman discharged on day 3 with a second baby.

On average, each home visit was 29 min (range 20 to 50 min) and phone calls (instead of home visit) were on average 9 min (range 7.5 to 11 min). Eight of the 15 women did not receive home-based midwifery care and commented that they were disappointed about that.

I didn't have anyone from the hospital come home. I think they should start introducing that, at least one visit, not to go overboard, just one, see how you're going and then the family healthcare (CFH) nurses to come when they should come. (M1, Para 1, interview).

The services provided during the midwifery visits at home were the same as the care provided on the ward. Each woman recorded that she had a physical check by the midwife (indicated in the blue box in supplementary files 2 to 6) and the baby was checked (green in files 2 to 6) and routine screening tests were done on specified days. M11 who had a caesarean section reported the care activities in her interview: “They come, and they measured weight. They check the wound, the baby, and everything” (M11, para 2). The physical health check of mother was often undertaken via a conversation with the woman rather than, for example, looking at her perineum.

#### Women’s perceptions of the home-based midwifery service

Women who received the home-based midwifery rated this service in their diary as very helpful, stating that the home visits from the midwife were reassuring and made them feel more comfortable about being home with their baby. Women made several comments in their diary about home-based midwifery care:

It made me feel like I wasn’t alone, like I do have that extra support and I could ask any questions that I had…they came to visit.. I got phone calls every couple days, I had a number to contact if I needed to. (M2, Para 1, interview).

I actually thought that there was actually more support going home than there was at the hospital…between the visits there were little calls as well, so it was always, every day was someone sort of checking in (M13, Para 1, interview).

Following discharge from the maternity service, or even while still being seen by midwives at home, 14 women received a home visit from the CFHN service (see supplementary files 2 to 6). One woman who had her second baby declined the CFHN visit stating she would go to her GP. The home visit was conducted when the babies were between 6 and 11 days of age and on average were 67 min long (45 to 90 min). In some instances, these visits overlapped with a visit from the midwife. The prime reason that women attended their GP was for a recommended check of the baby’s heart at around 7 days postnatal. Women were mostly very satisfied with the CFHN service they received in the home. Women’s comments reflected the value of information giving, the reassurance and positive appraisal they received from nurses and the assistance with specific tasks such as breastfeeding.

The CFHN talked us through all the things that she was looking for when she looked at [baby]. She made me feel quite at ease (M9, Para 1, interview).

The nurse spoke to me about quite a lot of things that I didn't actually get during my time in the hospital. like the way baby should be sleeping. the best way that the baby should be put down and not her tummy and not high up in the cot and things like that. Nobody actually talked to me about it. Things like the pelvic floor exercises, nobody, again, told me about things like that. She left some brochures and told me about things, what to do (M6, Para 1, interview).

## Discussion

In this study we used activity diaries completed by 15 women to map the mother-baby postnatal journey in one hospital in NSW, Australia. to describe the care provided to women and their babies from birth to discharge from the maternity service and identify possible service gaps. The findings show that these participants and their babies received limited direct /face-to-face postnatal care from midwives and other health professionals in hospital. In general, both primiparous and multiparous participants were satisfied with their hospital LOS and the care they received but indicated where improvements are needed. Those who did not receive home-based midwifery care were disappointed, suggesting that women want more assistance in the early postnatal period. Some women received multiple service encounters in proximity, for example, a midwife visiting one day, followed by a visit to the GP and then a home visit from the CFHN on consecutive days. The variable pattern of service delivery has been identified in other Australian studies [38, 39] This previous research indicates that some families use multiple providers for similar needs and others may not access services at all.

The average postnatal LOS in hospital in this study approximates with the current Australia-wide LOS [5]. The LOS post birth varies widely across and within high income countries ranging from 6 to 72 h and longer for women with complications or in private hospitals [40, 41]. There is no clear direction on the optimal postnatal LOS, and this may differ for mother and baby. A recent systematic review on the outcomes of early discharge [42] revealed that infants discharged, at or before 48 h after vaginal birth and, 96 h after caesarean birth, were more likely to be readmitted to the hospital within 28 days compared to standard discharge at around 72 h. A meta-analysis of readmission for neonatal jaundice found the risk of readmission decreased by 48% with every day added to the original hospitalization stay [43].

Studies have also reported that women having a caesarean section are more likely to be readmitted to hospital in the first week after discharge than women who had a vaginal births [44] but other population based studies suggest that readmissions have been higher for women when discharged on day one following a vaginal birth [45]. A recent review of studies examining women’s experiences of a reduced LOS after caesarean section suggests shorter LOS does not negatively impact on women, provided they are adequately prepared for discharge, are recovering well, and have continued pain relief and ongoing midwifery care at home [46]. These findings are supported by Cusack who emphasises that home visits by midwives are critical for women who had a caesarean Sect. [47]. Despite this discussion about the potential poorer outcomes if a woman and baby are discharged ‘early’ from hospital, it is rare that these studies specify if the mother and baby received a visit in the community from a midwife following discharge and, whether that makes a difference to outcomes [42].

Furthermore, determining whether a mother and her infant fare better if they have 6, 36, 48 or 72 h in hospital says little about how much and what care is provided during that time. In another study undertaken by this team, we found women who gave birth in private hospitals (who have longer in hospital stays) had more admissions to residential parenting services for sleep, feeding and psychological support [21]. These women tend to have little to no community-based midwifery postpartum support programs following discharge home. Iinterestingly, we found only 4.3% of women’s time in the postnatal unit (i.e. approximately one hour per 24 h) was spent in direct face-to-face interaction with a midwife and or doctor. While not expressed as a percentage of total LOS previously, others have reported the limited amount of one-to-one time that a woman receives from a midwife, noting that care interactions without interruption are typically less than 5 min, and 10 min duration when including an assessment of mother or baby [3, 6].

In this study, the 3 h of direct in-hospital care delivered prioritised the physical health of mother and baby over emotional care and information on long term health needs. As per local and state guidelines, physical postnatal ‘checks’ of mother and baby were conducted at least once in each 24-h period. Research conducted in Victoria [48] and Western Australia [9] a decade or more prior to our study, also indicated women are more likely to receive physical assessment with limited opportunities for assessment of, or discussion about, psychosocial needs or emotional support in hospital following birth. Yet, for well over a decade, women have reported feeling overwhelmed by the responsibilities being the primary caregiver for the newborn and having to attend to her own self-care needs while recuperating from birth [49, 50].

We found that in the first 12 to 24 h few women in this study received support for breastfeeding other than a 5 to 10-min period of instruction. The support for breastfeeding increased on day two, with women noting more frequent interactions around breastfeeding between 5 and 20 min and some received support from a LC. Limited breastfeeding support in hospital, at home or in the community is a common concern reported by new mothers [29, 50].

Researchers in the UK also reported that during the postnatal stay in hospital, women received an average of 75 min of care or education and feeding advice [51]. They also found this care was mostly delivered during the admission and discharge processes in the postnatal unit, As mentioned by the participants in our study, women often comment that they must wait a long time to get assistance when needed. Bowers and Cheyne noted that much of midwives’ and other staffs’ time is directed to some non-patient related tasks, for example, completing documentation, taking them away from the ‘emotion work’ of direct care [40]. Two studies have reported initiatives designed to increase the amount of time that a midwife spends with a woman, by redesigning aspects of care including the provision of a parenting room staffed by a midwife [52, 53].

Differences between length of stay and interaction time particularly related to breastfeeding were observed between primiparous and multiparous participants. As reported in other studies [3, 54] primiparous women experienced a longer LOS than multiparous, although this did not appear to impact their experience or perception of care. Indeed, in this small sample, the same number of multiparous women received midwifery care at home as primiparous women. This is likely because they had a shorter LOS in the postnatal unit. First time mothers typically require more support with breastfeeding than women who have breastfed before. Lindblad et al. [54] examined the difference between LOS and breastfeeding in first time and subsequent mothers and found that primiparous women had a higher risk of having doubts about infant feeding after discharge and discontinuing breastfeeding than multiparous women.

Differences between length of stay and interaction time particularly related to breastfeeding were observed between primiparous and multiparous participants. As reported in other studies [3, 54] primiparous women experienced a longer LOS than multiparous, although this did not appear to impact their experience or perception of care. Indeed, in this small sample, the same number of multiparous women received midwifery care at home as primiparous women. This is likely because they had a shorter LOS in the postnatal unit. First time mothers typically require more support with breastfeeding than women who have breastfed before. Lindblad et al. [54] examined the difference between LOS and breastfeeding in first time and subsequent mothers and found that primiparous women had a higher risk of having doubts about infant feeding after discharge and discontinuing breastfeeding than multiparous women.

Despite the limited interaction with midwives in the postnatal unit, participants valued knowing that midwives were there to assist them when they needed it and appreciated seeing the same midwife across shifts. There were also aspects of care and the postnatal environment, or organisation of care, that women found unhelpful or distressing. This is consistent with recent reports of lower satisfaction within hospital postnatal care when compared with other aspects of maternity care [55, 56]. Respondents in previous surveys of postnatal care have been less positive about the availability of the midwives, emotional care and information on maternal health needs and consistency of advice [39, 49, 57]. In general, first time mothers have previously rated both the style and quality of care more negatively than multiparous women [9].

In contrast, women are consistently more satisfied with midwifery care at home than in hospital [7, 9]. It is interesting therefore that the prime reason women in Australia [58], and elsewhere, given for staying in hospital is access to 24 h support [59]. When presented with a number of options for home based care many women still indicate their preference for a longer hospital stay, not just in Australia [58, 60], but also in Scandinavian countries [61] and in Switzerland [62]. This reflects the need, particularly for first-time mothers, for 24-h professional support. Indeed women report feeling pressured to leave hospital before they felt ready, and not having enough time to rest and recover [60]. Although there appears to be limited or no correlation between length of hospital stay and satisfaction with the postnatal experience [51].

The home-based midwifery program in this study site aimed to provide postnatal care for up to 2 weeks after birth for all eligible new mothers, however, on average, only 50% of women, both primiparous and multiparous, were offered a home visit at this time. This is lower than the proportion who received one home visit in Victoria (76%) and South Australia (88%) [63]. The National Institute of Clinical Excellence (NICE) guidelines [64] recommend a minimum of 3 home contacts post-discharge and WHO recommend 3 postnatal face to face contacts following an initial visit after birth by a health professional with the appropriate skills and competencies [1]. This limited home-based postnatal care contrasts with the postnatal care to six weeks offered by privately practising midwives in Australia and midwifery care in New Zealand where midwives are funded to provide a minimum of 5 and up to 10 home visits in the 6 week period following birth [65] It is unknown if this extended postnatal care results in better outcomes, including less readmissions. The postnatal midwifery care delivered at home mirrored that provided in hospital, as it was guided by the routine postnatal care pathway. Forster et al. (2016) report that there is a general uniformity regarding maternal and neonatal observations undertaken during a home visit [66]. Despite the focus on physical care of mother and baby, a recent systematic review [7] demonstrated that women hold home-based care in high regard and that it provides an opportunity to support women in the transition to motherhood in the early postnatal period.

### Practice implications

While guidelines for postnatal care are available in the UK [64], Australia currently lacks a national postnatal care guideline, and this is a potential barrier to providers implementing high-quality postnatal care [67]. It remains unclear how much postnatal care is required both in hospital and in the community to make a positive difference to outcomes for women. CFHN and other professionals have raised concerns about the limited amount of support that women receive in hospital and the limited service capacity to provide ongoing support to women, particularly for breastfeeding [3, 6].

Staffing is consistently identified as the major barrier to providing adequate one-to-one care in postnatal wards [6]. There are significant issues related to inadequate staff/patient ratios; staffing mix; patient mix; prioritisation of birth suite requirements over postnatal units; and the use of non-permanent staff. In some cases, care in postnatal wards will be provided by non-registered midwives typically registered nurses. There is also concern that reducing the LOS for women postnatally may compromise the care they receive and increase the amount of community-based postnatal care required [51]. It is noted that staff workload increases with a shorter LOS because staff complete all admission and discharge processes within severe time constraints, leaving little time to implement the care women perceive they need and expect they will [6, 51] receive from their in-patient stay, such as advice on how to care for their babies. Research in the UK has noted that the majority of postnatal care time is devoted to admission and discharge [51]. All of this became increasingly pressured and restricted during the COVID-19 pandemic.

Maternity services and health professionals also need to be aware that women’s needs for support in the postnatal period are diverse and LOS or number of visits that women require vary. McLeish et al. (2020) identified five postnatal care trajectories, showing that mothers’ satisfaction with postnatal care and confidence were primarily influenced by the extent to which they perceived their individual postnatal needs were met. They recommended that rapid and responsive assessment of needs both antenatally and postnatally, and appropriate adjustment of care, are key in supporting women effectively at this time [50].

Some authors have reported on studies attempting to change the delivery of postnatal care to meet women’s individual needs. We note that the more effective initiatives appear to be those that made changes across the organisation following an all-of-service continuous quality improvement project [34, 68]. Some services, notably in Scandinavian countries, have trialled approaches using telemedicine to provide ongoing contact with health professionals following discharge from a maternity unit [69]. During the recent COVID-19 pandemic telehealth has increased, likely contributing more to future postnatal care. In general, however evidence-based guidelines for postnatal care have not been implemented in practice, particularly the requirement for individualised care planning.

### Limitations

There are several limitations of this study. First the findings are based on the diary recordings and perceptions of only 15 women, 8 primiparous and 7 multiparous, who gave birth in one maternity service in NSW, Australia. It is possible that participants did not complete diary entries at the time of the interaction and recall bias may have impacted what was documented, Participation was limited to those who were able to read and write in English and this has excluded an important population group who are potentially more vulnerable particularly if they receiving limited postnatal care. The diaries also did not capture the postnatal care provided in the delivery suite. Therefore, the findings may not be generalisable to other settings in NSW or Australia where staffing and resource allocations and models of care may differ. Following the COVID-19 pandemic postnatal care may have altered in ways that will impact on future service provision, such as the increased use of telehealth.

## Conclusion

This study has focused on the quantity and nature of postnatal care in hospital and at home prior to discharge from the maternity service. It suggests that some women, both primiparous and multiparous, receive limited support from health professionals, both in hospital and at home. Reported shortening of in-hospital stays combined with low satisfaction with postnatal care has led to an increased concern as to whether services can adequately meet the needs of women following birth and that this is placing increased pressure on community based and ancillary services. In this study women reported that staff on the postnatal ward were rushed and mostly the midwife caring for them was unfamiliar to them. Women who received postnatal care at home were more satisfied with this care and those who did not receive home-based care were disappointed. Breastfeeding in the first 12–24 h was limited, with women wanting more one-on-one access to midwives. Home based midwives were more likely to engage women in conversations about their social and emotional needs.

## Supplementary Information

Supplementary Material 1.

Supplementary Material 2.

Supplementary Material 3.

Supplementary Material 4.

Supplementary Material 5.

Supplementary Material 6.

## Data Availability

Data is provided in supplementary files in graphical form. Additional data can be accessed form the authors upon a reasonable request.

## Abbreviations

CFHN: Child and Family Health Nurse
GP: General Practitioner
LOS: Length of Stay
NICE: National Institute of Clinical Excellence
UK: United Kingdom
WHO: World Health Organization
