# Supplementary material for: A mixed methods study of the postnatal care journey from birth to discharge in a maternity service in New South Wales, Australia
Source: BMC Health Serv Res. 2024 Dec 3;24:1530. doi: 10.1186/s12913-024-11995-w (PMC11613488; doi:10.1186/s12913-024-11995-w)
Supplement: Supplementary file 1 — Supplementary Material 1. [file 12913_2024_11995_MOESM1_ESM.pdf]

POPPY ACTIVITY DIARY - HOSPITAL

Please let us know about the care provided to you and your baby every time you have an interaction at the hospital

Date: **My baby is_____hrs/days/weeks old**

What time of day did the person come to you or your baby?	Who came?	How long was the interaction?	Why did they visit you/ enter the room? (please tick from below)				
			My health	My baby's health	Breastfeeding support /education	Discharge Information	Other:
☐ 6 am – 8 am<br>☐ 8 am – 10 am<br>☐ 10 am – 12md<br>☐ 12md – 2 pm<br>☐ 2 pm – 4 pm<br>☐ 4 pm – 6 pm<br>☐ 6 pm – 8 pm<br>☐ 8 pm – 10 pm<br>☐ 10 pm – 12mn<br>☐ Overnight 12mn to 6 am	○ Midwife (familiar)<br>○ Midwife (unfamiliar)<br>○ Student midwife<br>○ Doctor<br>○ Senior Dr<br>○ Junior Dr<br>○ Paediatrician/baby doctor<br>○ GP<br>○ Lactation Consultant<br>○ Hearing technician<br>○ Blood collector<br>○ Social Worker<br>○ Mental Health Professional<br>○ Physiotherapist<br>○ Housekeeping<br>○ Visitors/Friends/ Family<br>○ Other	Approximately<br>○ >5 mins<br>○ 5 mins<br>○ 10 mins<br>○ 15 mins<br>○ 20 mins<br>○ 25 mins<br>○ 30 mins<br>○ 30 - 45 mins<br>○ 45 - 60 mins<br>○ >60 mins	○ Taking my temperature, pulse, blood pressure<br>○ Blood tests<br>○ Checking my body (eg breasts, belly, stitches)<br>○ Talking with me about how I feel (my emotions)<br>○ Medications (e.g. pain relief)<br>○ Supporting me with personal hygiene (e.g. showering)<br>○ Providing education about my health<br>○ My paperwork<br>○ Referral to a service / other person<br>○ Other:	○ Blood tests<br>○ Weighing baby<br>○ Warming up baby<br>○ Immunisation (e.g. Hepatitis B)<br>○ Bowel movements<br>○ Passing urine<br>○ Jaundice<br>○ Umbilical cord care<br>○ Baby bathing<br>○ Sleep, settling / crying<br>○ General baby care advice<br>○ Baby's paperwork<br>○ Referral to a service / person<br>○ Other:	○ Breastfeeding support /education<br>○ Formula feeding assistance/education<br>○ Expressing breastmilk education/support<br>○ Breastfeeding support group<br>○ Other:	○ Given blue book for my baby, SIDS, safe sleeping, baby capsule<br>○ Information prior to discharge (e.g. contraception, physical signs to watch for)<br>○ Information about:<br>○ Community services e.g. child & family health nurse<br>○ Local GP<br>○ Breastfeeding support groups<br>○ Other services<br>○ Other:	○ Meal: Morning/ afternoon tea delivered<br>○ Meal: delivered and/or meal tray removed<br>○ Made own breakfast in kitchen<br>○ Cleaning services<br>○ Caring for other women / babies in room<br>○ Staff Looking for other staff<br>○ Other:

**How helpful was this?**	☐ Very unhelpful ☐ Unhelpful ☐ Neither helpful nor unhelpful ☐ Helpful ☐ Very helpful
**Since my last entry I sourced information about my baby and my health from:**	◻ General health website ◻ Government websites ◻ Online forums ◻ Family / friends ◻ Other
**NOTES / COMMENTS** (also see over page)	

ACTIVITY DIARY – HOME/COMMUNITY
Please let us know about the care of you and your baby every time you receive care at home/in the community

Date: **Day of week** **My baby is______days/weeks old**

What time of day did the person visit?	Who was this contact with?	How long was the interaction	Why did they visit you/ enter the room? (please tick from below)		
			**My health**	**My baby's health**	**Feeding my baby**
☐ 8 am – 10 am<br>☐ 10 am – 12md<br>☐ 12md – 2 pm<br>☐ 2 pm – 4 pm<br>☐ 4 pm – 6 pm<br>☐ 6 pm – 8 pm<br>☐ 8 pm – 10 pm<br>☐ Overnight 10pm to 8am	Midwife<br>○ Telephone call<br>○ First home visit<br>○ Follow up visit<br>○ Went to hospital to see midwife<br>Child and Family Health Nurse<br>○ First home visit<br>○ Follow up home visit<br>○ Telephone call<br>○ Went to clinic for visit<br>○ Saw nurse at mothers' group<br>○ 24 hour telephone helpline<br>○ Parent / mother group through church or other community group<br>○ My family GP<br>○ A new GP<br>○ Paediatrician<br>○ Obstetrician<br>Australian Breast Feeding Association<br>○ website/Facebook page<br>○ ABA group<br>○ ABA telephone helpline<br>Other professionals in community<br>○ Lactation Consultant in community<br>○ Lactation consultant at hospital<br>○ SWISH hearing screener<br>○ Blood collector<br>○ Other: e.g. social worker, naturopath	Approximately<br>○ >5 mins<br>○ 5 mins<br>○ 10 mins<br>○ 15 mins<br>○ 20 mins<br>○ 25 mins<br>○ 30 mins<br>○ 30 - 45 mins<br>○ 45 - 60 mins<br>○ 1 to 2 hours<br>○ > 2hours	○ Talking with me about how I feel (my emotions) or my worries or about my birth<br>○ Talked with me about my relationship with my partner<br>○ Talked with me about my family<br>○ Talking with me about being a parent<br>○ Taking my temperature, pulse, blood pressure<br>○ Blood tests<br>○ Checking my body (eg breasts, belly, stitches)<br>○ Medications (e.g. pain relief)<br>○ Providing education about my health<br>○ My paperwork/ computer<br>○ Referral to a service / other person<br>○ Other:	Providing information about<br>○ Baby's growth & development<br>○ Bowel movements, passing urine<br>○ jaundice<br>○ Umbilical cord care<br>○ Baby bathing<br>○ Sleep, settling / crying<br>○ Checking my baby – eg. weighing measuring baby; reflexes; vision.<br>○ Blood tests<br>○ Immunisation<br>○ Baby's paperwork/computer<br>○ Referral to a service<br>○ **Other**	○ Breastfeeding support /education<br>○ Formula feeding assistance/education<br>○ Expressing breastmilk education/support<br>○ Breastfeeding support group<br>○ Other:

**How helpful was this?**	◻ Very unhelpful ◻ Unhelpful ◻ Neither helpful nor unhelpful ◻ Helpful ◻ Very helpful
**Since my last entry I sourced information about my baby and my health from**	◻ General health website ◻ Government websites ◻ Online forums ◻ Family / friends ◻ Other

NOTES / COMMENTS (also see over page)
