# Supplementary material for: A mixed methods study of the postnatal care journey from birth to discharge in a maternity service in New South Wales, Australia
Source: BMC Health Serv Res. 2024 Dec 3;24:1530. doi: 10.1186/s12913-024-11995-w (PMC11613488; doi:10.1186/s12913-024-11995-w)
Supplement: Supplementary file 2 — Supplementary Material 2. [file 12913_2024_11995_MOESM2_ESM.pdf]

MOTHER 2: 23 year old, first baby, living with parents and boyfriend, stayed in hospital for 3 days, had 2 visits from CFHN, then only used GP care

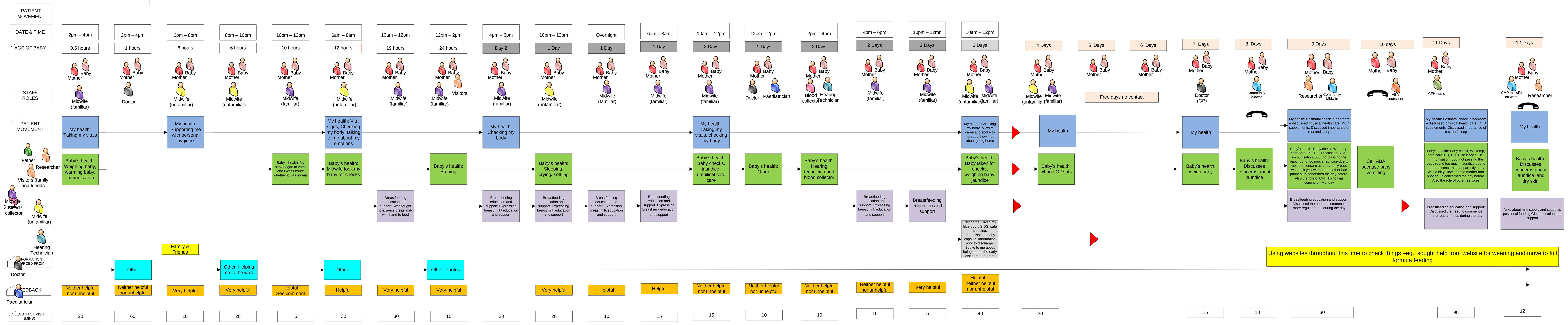

Title

PATIENT MOVEMENT

STAFF ROLES

PROCESSES

INFORMATION CREATION/ UPDATE (medium)

PATIENT NEEDS/ CLINICAL GUIDELINES/ POLICIES

MEASURE-MENTS
