# Supplementary material for: A mixed methods study of the postnatal care journey from birth to discharge in a maternity service in New South Wales, Australia
Source: BMC Health Serv Res. 2024 Dec 3;24:1530. doi: 10.1186/s12913-024-11995-w (PMC11613488; doi:10.1186/s12913-024-11995-w)
Supplement: Supplementary file 3 — Supplementary Material 3. [file 12913_2024_11995_MOESM3_ESM.pdf]

MOTHER 4: 29 years - first baby – LOS – 64 hours – High community service use for anxiety- receives midwifery @ home, CFHN, social workand GP

DATE & TIME	AGE OF BABY	PATIENT MOVEMENT / STAFF ROLES	PATIENT MOVEMENT					FEEDBACK	INFORMATION SOURCED FROM	LENGTH OF VISIT (MINS)
Day 1 12mn – 6am	1 hours	Mother, Baby, Midwife (familiar)	My health: Checking my body, supporting me with personal hygiene					Very helpful		10
Day 1 12mn – 6am	2 hours	Mother, Baby, Senior Doctor, Midwife (familiar), Doctor		Baby's health: Took my baby for checks (neither helpful..) and came back for results (helpful – see comments)				Neither helpful nor unhelpful/ helpful		10
Day 1 6am – 8am	6/7 hours	Mother, Baby, Midwife (unfamiliar), Housekeeping	My health: Taking my vital signs				Cleaning services	Very unhelpful (cleaning) Helpful (midwife)		10
Day 1 8am – 10am	7 hours	Mother, Baby, Housekeeping					Meal delivered			5
Day 1 10am – 12md	8 hours	Mother, Baby, Midwife (unfamiliar), Housekeeping	My health: Taking my vitals, checking my body, medications, providing education about my health	Baby's health: General baby care			Other	Very helpful		10
Day 1 12md– 2pm	8 hours	Mother, Baby, Father, Midwife (familiar), Housekeeping	My health: Medications, providing education about my health				Meal delivered	Helpful		5
Day 1 2pm– 4pm	12 hours	Mother, Baby, Father, Midwife (familiar)	My health: Talking with me about how I feel, medications	Baby's health: General baby care	Breastfeeding: See comment		Meal delivered	Helpful (midwife) Very helpful (meal)	Family , Friends, general health websites	5
Day 1 4pm– 6pm	12 hours	Mother, Baby, Father, Midwife (familiar), Midwife (unfamiliar)	My health: Taking my vitals, checking my body, talking with me about how I feel, medications	Baby's health: General baby care	Breastfeeding education and support		Made own dinner	Very helpful	Family , Friends, general health websites, online forums	25
Day 1 6pm– 8pm	14 hours	Mother, Baby, Father								
Day 1 8pm– 10pm	18 hours	Mother, Baby, Midwife (familiar)	My health: medications		Breastfeeding education and support			Very helpful (Bfeeding), Medications (unhelpful)		15
Day 1 10pm– 12mn	22 hours	Mother, Baby, Midwife (familiar)	My health: medications		Breastfeeding education and support			Very helpful		10
Day 2 overnight	Day 2	Mother, Baby, Midwife (unfamiliar), Midwife (familiar)	My health: Talking with me about how I feel, providing education about my health		Breastfeeding education and support			Helpful -See comment		15
overnight	Day 2	Mother, Baby, Midwife (familiar)	My health: Talking with me about how I feel, providing education about my health		Breastfeeding education and support			Helpful – See comment		5
overnight	Day 2	Mother, Baby, Midwife (familiar)			Breastfeeding education and support			Very helpful		10
6am-8am	Day 2	Mother, Baby, Housekeeping					Housekeeping x 3	Helpful See comment	General health website	5
6am – 8am	Day 2	Mother, Baby, Midwife (familiar)	Medications					Very Helpful		5
8am – 10am	Day 2	Mother, Baby, Midwife (familiar)	My health: Taking my vitals. Talking with me about how I feel			Information prior to discharge	Housekeeping Meal delivered	Very Helpful/ helpful Discussing leavng. Lack of sleep		15
10am – 12md	Day 2	Mother, Baby, Paediatrician, Lactation consultant, Visitors (family and friends)	My health: Talking with me about how I feel, providing education about my health	Baby's paed check	Breastfeeding education and support, expressing breastmilk educations and support, breastfeeding support group			Very Helpful		40
12md – 2pm	Day 2	Mother, Baby, Midwife (familiar), Visitors (family and friends)	My health:Checking my body, talking with me about how I feel, providing education about my health, medications	Umbilical cord care and general baby care			Housekeeping Meal delivered/ made own lunch in kitchen	Helpful: Comment: Cant go home, no room on midwife program	General health website and online forums	15
2pm – 4pm	Day 2	Mother, Baby, Midwife (familiar), Visitors (family and friends)		Referral to social worker as she only works Tuesday and cant visit before I leave, she will call me next Tues			Housekeeping Meal delivered	Helpful		
4pm – 6pm	Day 2	Mother, Baby, Midwife (unfamiliar), Visitors (family and friends)	My health: Talking with me about how I feel, providing education about my health	Baby bathing				Helpful: See comment		15
6pm – 8pm	Day 2	Mother, Baby, Midwife (familiar), Visitors (family and friends)	Medications				Housekeeping Meal delivered/ made own breakfast in kitchen	Very Helpful		5
8pm – 10pm	Day 2	Mother, Baby, Midwife (familiar), Visitors (family and friends)	My health: Talking with me about how I feel					Very Helpful: see comment		10
10pm – 12mn	Day 2	Mother, Baby, Midwife (familiar), Visitors (family and friends)		Sleep, settling/ crying	Breastfeeding education and support			Helpful:Painful latch, said will be back to help breastfeed just has another patient to assist		20
overnight	Day 3	Mother, Baby, Midwife (familiar), Visitors (family and friends)	My health: Checking my body, medications, talking with me about how I feel, providing education about my health	Blood tests. 48 hour blood test (heelp prick) for baby in nursery. Sleep settling and crying. Lesson in wrapping baby			Housekeeping:	Very helpful: Checking stitches/ lots of pain (probably overtired) nurse caring and understanding, offered to take bub again.	Online forums	20
6am – 8am	Day 3	Mother, Baby, Housekeeping, Midwife (familiar), Visitors (family and friends)	My health: Taking my vitals, medications, talking with me about how I feel, providing education about my health			Information prior to discharge	Housekeeping: Again coming in curtains because baby is crying and commenting	Very Helpful: Discharge chat, panadoll. Housekeeping – unhelpful as per above comment but meals delivered - helpful		5
8am – 10am	Day 3	Mother, Baby, Doctor, Junior Doctor, Visitors (family and friends), Hearing Technician	My health: Checking my body, talking with me about how I feel, providing education about my health	General baby care. Referral to a service, hearing test		Information prior to discharge	Housekeeping: Cleaner sings up and down the hall – some people are trying to sleep	Very Helpful: Able to get early discharge program and go home		45
10am – 12md	Day 3	Mother, Baby, Midwife (familiar), Visitors (family and friends), Lactation consultant	My health: Taking my vitals, providing education about my health		Breastfeeding education and support	Information prior to discharge		Very Helpful		25
2pm – 4pm	Day 3	Mother, Baby, Doctor, Visitors (family and friends)	My health: Checking my body, my paperwork, referral to a service			Information prior to discharge		Very Helpful		10
8am – 10am 10am – 12md	Day 4	Mother, Baby, Midwife Telephone call	My health: Talking to me about their service, being a parent	Baby's health: babys general health				Helpful – Health chat Neither helpful nor unhelpful. Call to say coming to visit today/ another call 20 mins before visit	Family and Friends/ General health websites/ Online forums	20
10am – 12md	Day 5	Mother, Baby, Midwife Telephone call, Midwife First home visit	My health: Talking to be about their service, how I feel, my relationships etc. being a parent, checking my body, about vaginal loss, medications, my health, my paperwork, using personal health record, referral to a mothers group	Baby's health: Babys general health, bowel movements, umbilical cord care, babys growth and dev	Breastfeeding education and support. Expressing breastmilk			Very helpful. See comments Phoned to make appt to check baby	Online forums	30
2pm – 4pm	Day 5	Mother, Baby, Lactation consultant			Breastfeeding education and support. Expressing breastmilk			Very helpful. See comments	Online forums	10
6pm – 8pm	Day 5	Mother, Baby, Midwife Telephone call			Breastfeeding education and support. Expressing breastmilk			Very helpful.ABA website is great. Clear, helpful info and easy to navigate	Online forums	5
6pm – 8pm	Day 6	Mother, Baby, Midwife Telephone call						Helpful. Visit organised for tomorrow		5
10am – 12md	Day 7	Mother, Baby, Midwife Telephone call						Helpful. Nurse calls x 2 to say coming today		5
12md – 2pm	Day 7	Mother, Baby, Midwife Follow up visit	My health: Talking to be about family, my relationships etc. checking my body, referral to a service					Helpful. See comments		25
2pm – 4pm	Day 7	Mother, Baby, Midwife Went to hospital	My health: checking my body					Helpful. See comments	Online forums and general health website	Greater than 2 hours
8am – 10am	Day 8	Mother, Baby, Midwife Telephone call						Helpful. Called to say nurse coming today		5
2md – 2pm	Day 9	Mother, Baby, Child and Family Health Nurse		Baby's health: Babys general health, bowel movements, jaumdice, umbilical cord care, babys growth and dev, comunication, baby bathing, sleep settling, checking my baby, immunisation, 1-4 week blue book check	Breastfeeding education and support.			Very helpful. See comments	Family and friends/ general health websites/ gov't websites/ online forums	75 mins
10am – 12md	Day 10	Mother, Baby, Paediatrician, New Doctor		Baby's health: Babys paperwork, 1-4 week blue book check	Breastfeeding education and support.			Very helpful. See comments		20
12md – 2pm	Day 10	Mother, Baby, Midwife Follow up visit, Midwife Telephone call	My health: checking my body	Baby's health: Babys papwerwork, 1-4 week blue book check	Breastfeeding education and support.			Unhelpful. See comments		25
2pm – 4pm	Day 10	Mother, Baby, Lactation consultant Phone call			Breastfeeding education and support.			Helpful. Made appt for Friday @ community centre Hoxton Park		5
2pm – 4pm	Day 10	Mother, Baby, Social worker	My health: Talking to me about their services, referral to another srevice					Very helpful. See comments		10
10am – 12md	Day 11	Mother, Baby, Midwife Telephone call	My health: Talking to me about their services, referral to another srevice					Very helpful. See comments	general health websites/ gov't websites/ online forums	10
10am – 12md	Day 13	Mother, Baby, Lactation consultant In community	My health: Talking to me about their services, referral to another service		Breastfeeding education and support. Breast feeding clinic/ support			Very helpful. See comments	general health websites/ gov't websites/ online forums	45-60
8am – 10am	2 weeks	Mother, Baby, Midwife Telephone call	My health: Other					Helpful. See comments	Family and friends/ general health websites/ gov't websites/ online forums	5
2pm – 4pm	2 weeks	Mother, Baby, Social worker	My health: Talking to me about their services, how I feel, about my family etc, my health and safety					Very helpful. See comments	Family and friends/ general health websites/ gov't websites/ online forums	45-60

Blood collector
Visitors (family and friends)
Paediatrician
Hearing Technician
Senior Doctor

Title

PATIENT MOVEMENT

STAFF ROLES

PROCESSES

INFORMATION CREATION/ UPDATE (medium)

PATIENT NEEDS/ CLINICAL GUIDELINES/ POLICIES

MEASURE-MENTS
