# Supplementary material for: A mixed methods study of the postnatal care journey from birth to discharge in a maternity service in New South Wales, Australia
Source: BMC Health Serv Res. 2024 Dec 3;24:1530. doi: 10.1186/s12913-024-11995-w (PMC11613488; doi:10.1186/s12913-024-11995-w)
Supplement: Supplementary file 4 — Supplementary Material 4. [file 12913_2024_11995_MOESM4_ESM.pdf]

MOTHER 6: 30 years, first baby, LOS 57 hours, no midwifery at home, one visit back to hospital at 20 days for breastfeeding support, CFHN home visit and then visited CFHN in clinic 3 times and saw GP

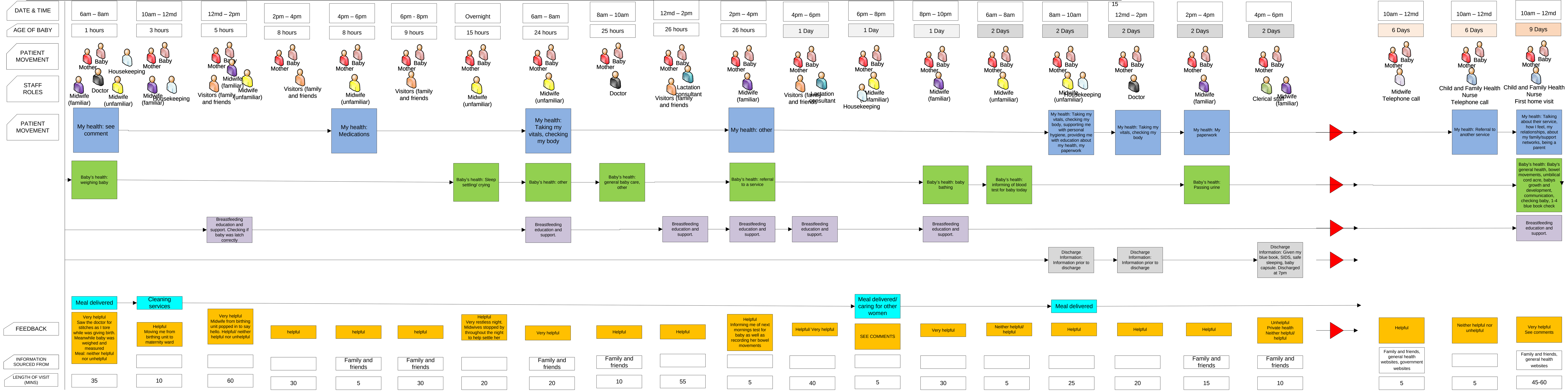

Title

PATIENT MOVEMENT

STAFF ROLES

PROCESSES

INFORMATION CREATION/ UPDATE (medium)

PATIENT NEEDS/ CLINICAL GUIDELINES/ POLICIES

MEASURE- MENTS
