# Supplementary material for: A mixed methods study of the postnatal care journey from birth to discharge in a maternity service in New South Wales, Australia
Source: BMC Health Serv Res. 2024 Dec 3;24:1530. doi: 10.1186/s12913-024-11995-w (PMC11613488; doi:10.1186/s12913-024-11995-w)
Supplement: Supplementary file 5 — Supplementary Material 5. [file 12913_2024_11995_MOESM5_ESM.pdf]

MOTHER 9: 29 years; first baby; LOS 66 hours, only received 14 mins breastfeeding support, no midwifery@ home, had one visit at home from CFHN, attended mothers' groups and GP

DATE & TIME	AGE OF BABY	PATIENT MOVEMENT	STAFF ROLES	PATIENT MOVEMENT					FEEDBACK	INFORMATION SOURCED FROM	LENGTH OF VISIT (MINS)
8am – 10am	5 hours	Mother, Baby	Midwife (familiar)	My health: Checking my body	Baby's health: Glucose test	Breastfeeding education and support			Helpful		15
8am – 10am	7 hours	Mother, Baby	Visitors (family and friends						Neither helpful nor unhelpful		60
12pm – 2pm	8 hours	Mother, Baby	Midwife (familiar)		Baby's health: Glucose test: Discussed options because of low glucose	Breastfeeding education and support			Helpful		5
12pm – 2pm	8 hours	Mother, Baby						Meal delivered/ tray removed	Neither helpful nor unhelpful		5
2pm – 4pm	10 hours	Mother, Baby	Midwife (unfamiliar)	My health: Temperature My paperwork	Baby's health: Baby's paperwork				Neither helpful nor unhelpful		5
2pm – 4pm	12 hours	Mother, Baby	Blood collector		Baby's health: Blood tests				Helpful She explained what she was doing		5
6pm – 8pm	12 hours	Mother, Baby	Midwife (familiar)		Baby's health: Glucose test						5
6pm – 8pm	13 hours	Mother, Baby	Visitors (family and friends						Neither helpful nor unhelpful		60
10pm – 12pm	14 hours	Mother, Baby	Midwife (unfamiliar)	My health: Glucose test results recording	Baby's health: Glucose test						5
Overnight - 6am – 8am	24 hours	Mother, Baby	Midwife (familiar)					Made own breakfast in kitchen	Neither helpful nor unhelpful		5
6am – 8am	1 Day	Mother, Baby	Housekeeping					Cleaning services	Neither helpful nor unhelpful		5
6am – 8am	1 Day	Mother, Baby	Midwife (familiar)					Made own breakfast in kitchen	Helpful		5
8am – 10am	1 Day	Mother, Baby	Midwife (familiar)	My health: Vital signs, checking my body, talking to me about how I feel & health education	Baby's health: Bowel movements, passing urine	Breastfeeding education and support			Helpful Explained what he was looking for e.g. seperation of mucles		10
8am-10am	1 Day	Mother, Baby	Midwife (unfamiliar)		Baby's health: Wanted to collect glucose sample	Breastfeeding education and support			Neither helpful nor unhelpful Misscommunication between midwives. Wanted to do test again even though it has been done		5
8am – 10am	1 Day	Mother, Baby	Hearing Technician		Baby's health: Hearing test				Helpful		5
12pm – 4pm	1 Day	Mother, Father, Baby	Midwife (familiar)		Baby's health: Umbilical cord care, baby bathing, general baby care				Very helpful Was happy to give my husband a turm		15
12pm – 2pm	1 Day	Mother, Baby	Housekeeping					Cleaning services	Neither helpful nor unhelpful		5
12pm – 2pm	1 Day	Mother, Baby	Housekeeping					Meal delivered/ tray removed	Neither helpful nor unhelpful		5
2pm – 4pm	1 Day	Mother, Baby	Visitors (family and friends						Neither helpful nor unhelpful		60
4pm – 6pm	1 Day	Mother, Baby	Visitors (family and friends						Neither helpful nor unhelpful		60
6pm – 8pm	1 Day	Mother, Baby	Housekeeping					Meal delivered/ tray removed	Neither helpful nor unhelpful		5
6am – 8am	2 Days	Mother, Baby	Housekeeping					Meal delivered Made my own breakfast in the kitchen	Neither helpful nor unhelpful		5
8am – 10am	2 Days	Mother, Baby	Midwife (unfamiliar)	My health: Vital signs, checking my body, talking to me about how I feel, medications & health education	Baby's health: Weighing baby						10
10am – 12pm	2 Days	Mother, Baby	Midwife (unfamiliar)	My health: Talking to me about how I feel health education & paperwork			Information prior to discharge. Information about local GP & breastfeeding support groups				15
12pm – 2pm	2 Days	Mother, Baby	Housekeeping					Meal delivered	Helpful		20
12pm – 2pm	2 Days	Mother, Baby	Paediatrician	My health: Talking to me about how I feel health education & paperwork	Baby's health: Weighing baby and discharge check up				Helpful		20
2pm – 4pm	2 Days	Mother, Baby	Midwife (familiar)	My health: Talking to me about how I feel health education & paperwork	Baby's health: Weighing baby and discharge check up		Given blue book for my baby, SIDS safe sleeping & baby capsule		Helpful		20
2md – 2pm	3 Days	Mother, Baby	Midwife Telephone call; Child and Family Health Nurse Telephone call		Baby's health: babys general health				Neither helpful nor unhelpful	General health websites	5
10am – 12md	6 Days	Mother, Baby	Child & Family Health Nurse First home visit	My health: How I feel, my relationship with my partner, about my familyect, being a parent, about vaginal loss, my health, using personal health record, referral to a mothers group	Baby's health: Babys general health, bowel movements, communication etc, checking my baby, 1-4 week blue book check				Helpful		15
8pm – 10pm	8 Days	Mother, Baby							Helpful	Online forums.GOOGLE: information on how much time she should be awkake	15
2pm – 4pm	10 Days	Mother, Baby	Family GP						Neither helpful nor unhelpful. Community nurse could not check babies heart beat. Need to go to the doctor.		5
2pm – 4pm	13 Days	Mother, Baby							Helpful. Googled to see why Sophie was choking while breast feeding	Online forums. GOOGLE	

Title

PATIENT MOVEMENT

STAFF ROLES

PROCESSES

INFORMATION CREATION/ UPDATE (medium)

PATIENT NEEDS/ CLINICAL GUIDELINES/ POLICIES

MEASURE-MENTS
