# Supplementary material for: A mixed methods study of the postnatal care journey from birth to discharge in a maternity service in New South Wales, Australia
Source: BMC Health Serv Res. 2024 Dec 3;24:1530. doi: 10.1186/s12913-024-11995-w (PMC11613488; doi:10.1186/s12913-024-11995-w)
Supplement: Supplementary file 6 — Supplementary Material 6. [file 12913_2024_11995_MOESM6_ESM.pdf]

MOTHER 15: 31 years old, second baby, LOS 22 hours, 3 midwifery home visits, one from CFHN and GP appointment

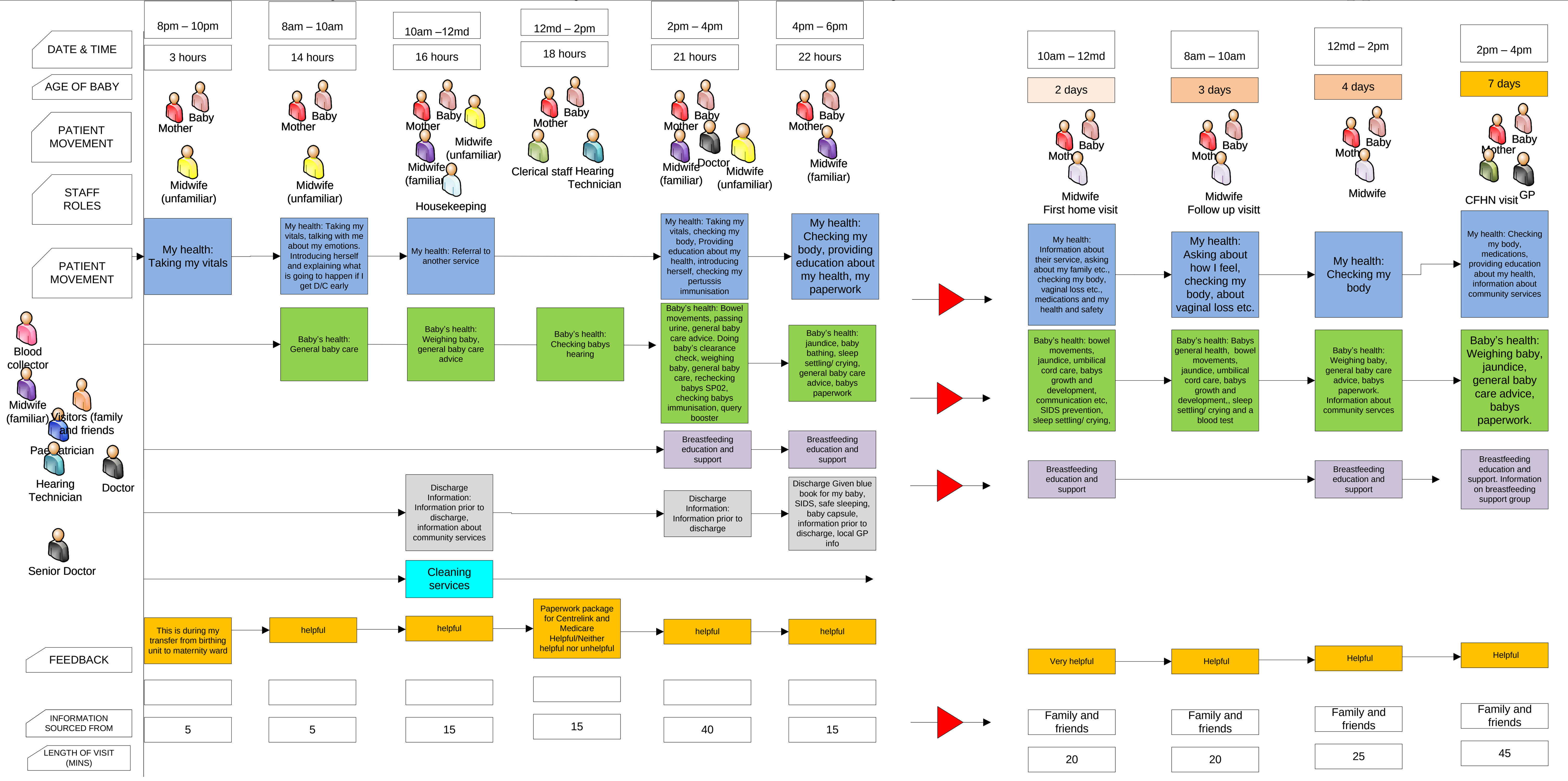

Title

PATIENT MOVEMENT

STAFF ROLES

PROCESSES

INFORMATION CREATION/ UPDATE (medium)

PATIENT NEEDS/ CLINICAL GUIDELINES/ POLICIES

MEASURE-MENTS
